# The impact of digital transformation of commercial banks on household income: Evidence from China

**DOI:** 10.1371/journal.pone.0310277

**Published:** 2024-09-13

**Authors:** Yuran Chen, Ruoxuan Huang, Yuying Zhang, Qinghong Shuai

**Affiliations:** 1 School of Finance, Southwestern University of Finance and Economics, Chengdu, Sichuan, China; 2 School of Business Administration, Southwestern University of Finance and Economics, Chengdu, Sichuan, China; 3 School of Finance, Yunnan University of Finance and Economics, Kunming, Yunnan, China; 4 School of Management Science and Engineering, Southwestern University of Finance and Economics, Chengdu, Sichuan, China; Zhengzhou University, CHINA

## Abstract

Scholars have focused on the digital transformation of commercial banks, yet there remains a lack of systematic and integrative research at the micro-level of household finance. This article uses data from the China Household Finance Survey (CHFS) and the Digital Transformation Index of Chinese Commercial Banks from Peking University. It employs empirical methods such as mechanism analysis and heterogeneity analysis to explore the impact of the digital transformation of commercial banks on household income. The findings indicate that the digital transformation of commercial banks significantly enhances household income. Second, increasing credit availability, fostering the development of digital inclusive finance, enhancing entrepreneurial possibilities, and increasing the purchase of wealth management products are key pathways through which digital transformation affects household income. Third, heterogeneity analysis reveals that the positive effects of digital transformation on household income are more pronounced in the central and western regions, areas with lower financial industry competition, regions with underdeveloped inclusive finance, rural areas, and among low-income families. This study highlights the significant role that the digital transformation of commercial banks plays in enhancing the welfare of the resident sector.

## 1. Introduction

Finance is central to the modern economy. The transformation and development of financial structures play a crucial role in the functioning of the global economy. As a critical component of the financial system, the banking industry influences both the reform and the deepening of financial development. In an information society driven by Information and Communication Technologies (ICTs), digital technology is transforming lives worldwide, emerging as a new driver of economic and social development (Barefoot et al., 2018; Cheng et al., 2021; Lin et al., 2023; Fuseini et al., 2024) [1–4]. In January 2022, the European Central Bank (ECB) released the “Guide to Digital Transformation of the European Banking Sector,” explicitly stating that Europe should resolutely pursue a development path reflecting its unique characteristics. The digital transformation of commercial banks should actively promote the digitalization of industrial finance and robustly advance the digital transformation of personal financial services. In January 2022, the Federal Reserve System of the United States published the “U.S. Digital Economy Development Plan,” proposing to expedite the incorporation of digital transformation in the financial sector as a key industry enhancement, promoting extensive use of big data, artificial intelligence, blockchain, and other technologies in banking and related fields. In October 2023, the International Monetary Fund (IMF) emphasized at the Global Financial Work Conference that prioritizing digital finance is crucial among five major tasks. Consequently, the digital transformation of banks is a pivotal focus for future financial reform and development globally, and a primary subject of financial development theories and policy research across various countries.

In China, the digital transformation of commercial banks is robustly supported by policy backing, technological innovation, market resources, and international competitiveness, providing a wealth of empirical data with significant academic value and practical implications. Specifically, First, the Chinese government prioritizes digital transformation highly and has implemented systematic planning. It has issued policy documents such as the “Guiding Opinions on Digital Transformation of the Banking and Insurance Industries” and the “14th Five-Year Plan for the Development of the Digital Economy,” which both advocate for accelerating digital transformation in the financial sector. Second, China’s rapid development and broad application of cutting-edge technologies, including big data, artificial intelligence, and blockchain, have established a solid technological foundation for the digital transformation of commercial banks. For example, using artificial intelligence in risk control, customer service, and business optimization has enabled Chinese commercial banks to achieve higher efficiency and enhance user experiences during their digital transformation (Lu et al., 2024) [5]. Third, China’s vast and diverse market offers a wide range of practical scenarios and data resources for the digital transformation of commercial banks. Through in-depth data analysis of customer behavior, banks can offer more precise and diverse financial products and services. Fourth, in terms of international cooperation, allowing entry to foreign banks and participating in the establishment of international standards have enhanced the international competitiveness of China’s banking industry. Through digital transformation, banks have introduced innovative products like mobile banking, smart advisory services, and blockchain-based cross-border payments, thereby improving customer experience and operational efficiency and promoting the development of inclusive finance.

Family formed through kinship and marriage, is a collective that lives together. It serves as both a fundamental unit participating in social activities and a crucial entity in making financial decisions (Himmelweit et al., 2013; Wang et al., 2023; Lin et al., 2023) [6–8]. Families, significant consumers of financial products, influence their financing and asset allocation through their financial behaviors, which in turn affects their levels of poverty and welfare (Wang and Li, 2023; Zehra and Singh, 2023) [9, 10]. Financial activity, encompassing cross-temporal and cross-spatial material exchange, inherently involves the optimal allocation of resources to maximize utility, with the banking sector playing a vital role. Furthermore, under modern market economy conditions, the banking sector, a crucial part of the financial industry, can significantly enhance family welfare levels.

Existing research serves as a reference for this paper, which primarily focuses on the theoretical aspects of digital transformation in the banking sector, including its essence, future prospects, and impact on macroeconomic factors. However, despite its critical role in household financial decisions, the significant impact of digital transformation in the banking sector on family welfare has received scant attention, with few studies empirically examining this impact. Consequently, this paper explores the effects of digital transformation in the banking industry on household income and distribution, aiming to enhance existing theories of financial development and household finance.

## 2. Literature review

A comprehensive review of existing literature shows that the digital transformation of commercial banks has attracted considerable attention from scholars. However, these studies primarily focus on the theoretical aspects such as the essence, key points, and development prospects of digital transformation in the banking sector (Baskerville et al., 2020; Naimi-Sadigh et al., 2022; Lu, 2023) [11–13]. It is suggested that the digital transformation of commercial banks involves the application of modern financial technologies to innovate financial services. Internally, this transformation facilitates shifting the banks’ value chain structure from a closed, self-circulating model to an open, collaborative model (Li and Zhang, 2024) [14], thus alleviating the conflict between financial inclusion and the profitability and safety objectives of commercial banks (Feghali et al., 2021) [15]. Externally, it helps reduce bank liquidity hoarding by enhancing information transparency and reducing the operational costs of credit operations (Diamond et al., 2001; Zhang et al., 2024) [16, 17], thereby improving bank performance, resisting the impact of new technologies, and facilitating channel transformation (Beccalli, 2007; Scott et al., 2017) [18, 19].

Only a few empirical studies have investigated the impact of digital transformation in the banking industry on bank risk and corporate development (Tsindeliani et al., 2022; Zhou and Li, 2023) [20, 21]. Jiang et al. (2023) empirically examined the risk-taking levels during the digital transformation of commercial banks, discovering that digital transformation significantly reduces management costs and enhances operational efficiency, thereby reducing risk-taking. Notably, the mitigating effect of digital transformation on risk-taking is more significant in smaller banks [22]. Zhang et al. (2021) argued that digital transformation in commercial banks significantly improves credit accessibility for small and medium-sized enterprises (SMEs), thereby enhancing the inclusiveness of financial services for these enterprises [23]. Especially in banks with a higher degree of digital transformation, improvements in cost efficiency and reductions in non-performing loan rates can alleviate the challenges of difficult and expensive loans faced by small and micro enterprises (Hakizimana et al., 2023) [24]. Furthermore, the digital transformation of banks significantly enhances corporate performance by alleviating financing constraints, and notably boosts the autonomous innovation capabilities of small and micro enterprises (Ahiadorme, 2018; Johri et al., 2024) [25, 26].

Existing research serves as both a reference and a benchmark for this paper. however, it is apparent that scholars have primarily concentrated on the essence, key aspects, and future prospects of digital transformation in the banking sector, as well as its impact on macroeconomic factors. Despite its significant influence on household financial decision-making, the profound impact of the banking sector’s digital transformation on family welfare has seldom been examined. Therefore, this paper investigates the effects of digital transformation in the banking industry on household income from the perspective of digital transformation, providing a valuable addition to existing theories of financial development and household finance.

The marginal contributions of this paper are threefold: First, in terms of conceptual transformation, this study broadens the research perspective from “banking finance” to the micro-level realm of “household finance.” Second, at the theoretical level, this paper analyzes the mechanisms and heterogeneous impacts of the digital transformation in the banking sector on household income and distribution from a micro-perspective. Third, in terms of practical significance, this paper extends the existing theories of “banking sector development” and “household finance,” providing significant real-world implications for enhancing household income.

The remainder of the content is organized as follows: The second section offers a theoretical analysis of the impact of digital transformation in commercial banks on household income and introduces this paper’s research hypotheses; the third section details the empirical testing and results analysis; the fourth section addresses the conclusions and provides policy recommendations.

## 3. Theoretical analysis and research hypothesis

Household income can be classified into wage income, business income, property income, and transfer income. Compared to high-income families, low-income families have fewer mortgage-eligible assets and lower property income. This disparity is especially pronounced in rural and other underdeveloped areas, where financial infrastructure is weak and some households lack credit records, resulting in a more homogeneous source of income. Therefore, given the income characteristics of low-income groups and the availability of financial resources, the digital transformation of commercial banks can primarily enhance household income through the following pathways.

### 3.1 Digital transformation of commercial banks, household credit accessibility, and family income

Amid financial constraints, impoverished groups find it challenging to access financial services due to low income levels, a lack of collateral assets, and no credit records. In contrast, wealthier groups, with their higher incomes, ample collateral assets, and solid credit histories, can easily secure high returns, further enhancing their wealth accumulation. Modern information technologies, such as big data and cloud computing, provide banks with low-cost, efficient tools for customer risk identification, effectively mitigating traditional financial exclusion issues. Through precise credit assessments and risk management, banks can better support their clients’ production and living needs, thereby facilitating household income growth (Jeník et al., 2020) [27].

Furthermore, the application of financial technology allows banks to significantly reduce credit costs. By employing more precise customer profiling and credit assessments, banks can offer credit services to creditworthy low-income groups who lack traditional credit support, thereby enhancing financial inclusivity. Additionally, by leveraging big data and other analytical technologies, banks can reduce credit risks, optimize credit processes, and enhance the availability and coverage of financial services. By increasing the efficiency of credit approval, banks also provide more families with the opportunity to access financial services, thereby aiding in the economic improvement and social advancement of impoverished households.

Therefore, the first research hypothesis of this paper is proposed:

H1: The digital transformation of commercial banks alleviates household credit constraints, thereby increasing household income.

Commercial banks, by adopting the “digital technology + inclusive finance” development model, effectively alleviate credit constraints and offer significant potential to narrow the urban-rural income gap and reduce income inequality (Xiong and Chen, 2020; Chen et al., 2024) [28, 29]. Specifically, this model not only enhances the overall “wealth level” of urban and rural areas but also strengthens the “sharing level” between them, achieving dual improvements in efficiency and equity (Wang et al., 2023) [30]. Guo et al. (2020) [31] noted that the application of digital finance in commercial banks, especially through innovations in credit approval and risk management, provides income-generating opportunities for farmers in economically backward areas. By lowering credit barriers, farmers in these areas can more easily access productive loans, thereby increasing their income, reducing income inequality, and fostering shared prosperity (Isa-Olatinwo et al., 2022) [32]. Do et al. (2022) found that the digital transformation of commercial banks significantly enhances intergenerational mobility and social equity. Digital tools such as mobile banking and online loan platforms not only simplify the process of obtaining financial services but also enhance the penetration rate of financial services, helping to improve families’ relative income levels and reduce economic vulnerability [33]. Particularly under the strong support of the Chinese government, digital inclusive finance significantly enhances family income, enabling more families to access convenient financial services and thereby improve their economic conditions (Xie and Wang, 2023) [34]. Furthermore, the enhancement of residents’ digital skills and increased usage of digital financial tools significantly elevate their income levels (Huang et al., 2023) [35].

Therefore, the second research hypothesis of this paper is proposed:

H2: The digital transformation of commercial banks enhances family income by facilitating the implementation of digital inclusive finance.

### 3.2 Digital transformation of commercial banks, family entrepreneurship, and family income

Entrepreneurship, as a principal source of family operational income, is influenced by multiple factors including the level of personal financial resources, the entrepreneurial environment, external information, and the accessibility of financial services (Halvarsson et al., 2018) [36]. In traditional economic societies, ordinary residents have limited means of accessing information, often relying on social networks. A lack of familiarity with commercial bank policies limits their grasp of financial market information. Moreover, traditional financial markets have high entry barriers, and many individuals, often lacking relevant financial knowledge and understanding of commercial bank products, cannot access necessary financial services. Issues such as high costs of information acquisition and financing constraints further hinder families from making entrepreneurial decisions and increasing their incomes (Song et al., 2020) [37]. With the rapid development of the Internet and information technology, the growth of the digital economy has significantly altered this situation. The information effect of the digital transformation of commercial banks has significantly reduced the costs for residents to acquire product and market information, helping them quickly understand market demands (Gao and Wang, 2023) [38].

Through digital platforms, residents can conveniently and swiftly access the latest market trends, policy information, and financial products, thus making more informed entrepreneurial decisions. Concurrently, the proliferation of online education and financial literacy campaigns has improved residents’ financial literacy and strengthened their capacity to utilize financial services for entrepreneurship.

Consequently, this paper proposes the third research hypothesis:

H3: The digital transformation of commercial banks increases family income by enhancing entrepreneurial opportunities for households.

### 3.3 Digital transformation of commercial banks, wealth management purchases, and household income

The digital transformation of commercial banks has transformed the traditional in-branch wealth management model, effectively reducing transaction costs for residents involved in financial wealth management and enhancing participation convenience (Khattak et al., 2023) [39]. Furthermore, household participation in financial markets contributes to increased property income and promotes upward income mobility among low-income groups. Yang et al. (2023) identified residents’ financial knowledge, financial accessibility, and transaction costs as significant factors that influence their participation in financial wealth management [40]. For low-income groups, high transaction costs and a lack of financial knowledge lead to a lower participation rate in the financial wealth management market, which impedes the increase in property income. Meanwhile, the development of the digital economy has promoted further integration of the internet and educational resources, altering the content, structure, and format of the knowledge supply. This transformation enables residents to update their financial knowledge at lower costs, and the dissemination of financial knowledge enhances residents’ awareness of wealth management and lending, breaking the constraints of insufficient knowledge on financial market participation.

Banks provide customized financial advice and products based on customers’ consumption behaviors and financial conditions, thus better meeting the diverse needs of different clients. This enhancement not only increases customer satisfaction but also improves the inclusivity and diversity of the financial markets. Furthermore, the widespread adoption of digital financial platforms significantly reduces the barriers to financial services, enabling more households to access professional wealth management services. This inclusive financial service model not only boosts residents’ property income but also fosters overall economic development and social equity.

Therefore, this paper proposes the fourth research hypothesis:

H4: The digital transformation of commercial banks enhances household income by increasing families’ purchases of wealth management and insurance products.

## 4. Empirical testing and result analysis

### 4.1 Data sources and description

Considering data availability and the accuracy of empirical results, the data were processed as follows:

First, the micro-level household data utilized in this study come from the “China Household Finance Survey (CHFS),” conducted by the Survey and Research Center for China Household Finance at Southwestern University of Finance and Economics from 2013 to 2019.

Second, the digital transformation index for commercial banks is sourced from the “Digital Transformation Index of Chinese Commercial Banks (2010–2021)” published by the Digital Finance Research Center at Peking University. This index is based on data from all commercial banks in China, with a final sample comprising 246 banks, including six major state-owned banks, 12 joint-stock commercial banks, 128 city commercial banks, 54 rural commercial banks, 29 foreign banks, and 17 private banks. The data for constructing the index are derived from annual reports of Chinese banks, patent data, and other sources. The data for constructing the index are derived from annual reports of Chinese banks, patent data, and other sources.

Additionally, the index includes three sub-dimensions: strategic digitalization, business digitalization, and management digitalization. Strategic digitalization refers to the importance banks place on digital technology. Business digitalization encompasses three levels: digital channels, digital products, and digital research and development. Management digitalization also includes three levels: whether there is a dedicated department responsible for digital transformation within the organizational structure, the presence of digital talent on the board and among senior management, and the extent of banks’ collaborative investments with technology companies. This index is frequently used by scholars domestically and internationally as a significant indicator of the digital transformation of commercial banks (Cao et al., 2022; Xie and Wang, 2023) [41, 42].

Third, the 2019 household agricultural production and business income data are sourced from the “Chinese Family Database (CFD),” constructed by the Social Science Research Platform and the Social Survey Research Center at Zhejiang University.

After excluding observations with missing key variables, a total of 125006 data points were obtained.

### 4.2 Model specification and variable definitions

To investigate the relationship between the digital transformation of commercial banks and household income, this paper establishes the following model:

$${total\_income}_{ijt}=\alpha_{0}+\beta_{1}{digital}_{t}+\beta_{2}X_{ijt}+\gamma_{t}+\lambda_{j}+\varepsilon_{ijt}$$ (1)

Where total_income_ijt_ represents the logarithm of the total household income for household i in city j at time t. The primary explanatory variable digital_t_ represents the total digitalization index of commercial banks at time t. X_ijt_ denotes control variables at the household, family, and regional levels. γ_t_ represents time fixed effects, λ_j_ represents regional fixed effects, and ε_ijt_ is the random error term.

Specifically, the first dependent variable is total household income. Households with negative total income values in the data from the China Household Finance Survey and Research Center were excluded, and the total household income was log-transformed after adding one. Second, the key explanatory variable is the degree of digital transformation of commercial banks. Third, control variables: Based on the studies of Khattak et al. (2023) [39], Xie and Wang (2023) [42], Chen et al. (2024) [29], and others, this research selects variables including characteristics of household heads, families, and regions.

Characteristics of the household head include: age, gender (male = 1, otherwise = 0), years of education based on survey responses (no schooling = 0, primary = 6, middle school = 9, high school = 12, vocational school = 13, junior college = 15, bachelor’s degree = 16, master’s degree = 19, doctoral degree = 22), health status using a scale (“Compared to others your age, how would you rate your health?” with ratings from very poor = 1 to very good = 5), marital status (married or cohabiting = 1, otherwise = 0), and employment status (employed = 1, otherwise = 0). Family characteristics include: household size, risk attitude (value 1 for preference towards high-risk high-return or moderate-risk moderate-return investments, otherwise 0), and total assets. Regional characteristics include: gross domestic product, public fiscal revenue, added value of the secondary industry, and added value of the tertiary industry. Table 1 provides descriptive statistics for the relevant variables.

**Table 1:** Descriptive statistics of key variables.

Variable	Name	Observations	Mean	Standard Deviation	Minimum	Maximum
Total Household Income	Ln(1+income)	125006	10.47	1.868	0	16.31
Overall Bank Digital Transformation Index	digital	125006	56.63	20.21	26.54	84.34
Age	age	125006	54.25	14.44	17	117
Gender	gender	125006	0.757	0.429	0	1
Married	married	125006	0.851	0.356	0	1
Years of Education	edcation	125006	8.273	4.297	0	22
Health Status	health	125006	3.213	1.073	0	5
Employed	work	125006	0.495	0.500	0	1
Risk Preference	risk	125006	0.0675	0.251	0	1
Household Size	household	125006	3.353	1.675	1	27
Total Household Assets	Ln(asset)	125006	12.66	1.798	1	21.47
Regional GDP	Ln(GDP)	125006	10.09	0.809	7.446	11.59
Public Fiscal Revenue	Ln(revenue)	125006	16.91	0.931	13.60	18.35
Added Value of Secondary Industry	Ln(secondary)	125006	9.166	0.891	6.524	10.68
Added Value of Tertiary Industry	Ln(thirdary)	125006	9.405	0.833	6.719	11.01

### 4.3 Results analysis

#### 4.3.1 Baseline regression results

Column (1) of Table 2 presents the effects of the overall Bank Digital Transformation Index on total household income. It shows that digital transformation in commercial banks leads to an increase in total household income.

**Table 2:** Commercial bank digitalization and household income.

	(1)	(2)	(3)	(4)
	Baseline Regression Results	Replacing the Explanatory Variable		
Overall Bank Digital Transformation Index	0.008***			
	(5.94)			
Strategic Digitalization Index		0.005***		
		(5.94)		
Business Digitalization Index			0.006***	
			(5.94)	
Management Digitalization Index				0.015***
				(5.94)
Age	0.002***	0.002***	0.002***	0.002***
	(3.51)	(3.51)	(3.51)	(3.51)
Gender	-0.098***	-0.098***	-0.098***	-0.098***
	(-7.73)	(-7.73)	(-7.73)	(-7.73)
Married	0.233***	0.233***	0.233***	0.233***
	(14.05)	(14.05)	(14.05)	(14.05)
Years of Education	0.026***	0.026***	0.026***	0.026***
	(22.54)	(22.54)	(22.54)	(22.54)
Health Status	0.094***	0.094***	0.094***	0.094***
	(18.44)	(18.44)	(18.44)	(18.44)
Employment	0.342***	0.342***	0.342***	0.342***
	(23.26)	(23.26)	(23.26)	(23.26)
Risk Preference	0.237***	0.237***	0.237***	0.237***
	(11.59)	(11.59)	(11.59)	(11.59)
Household Size	0.121***	0.121***	0.121***	0.121***
	(39.71)	(39.71)	(39.71)	(39.71)
Total Assets	0.348***	0.348***	0.348***	0.348***
	(93.75)	(93.75)	(93.75)	(93.75)
Regional Gross Domestic Product (GDP)	-0.180	-0.180	-0.180	-0.180
	(-0.42)	(-0.42)	(-0.42)	(-0.42)
Public Fiscal Revenue	-0.050	-0.050	-0.050	-0.050
	(-0.83)	(-0.83)	(-0.83)	(-0.83)
Secondary Industry Added Value	0.426**	0.426**	0.426**	0.426**
	(2.07)	(2.07)	(2.07)	(2.07)
Tertiary Industry Added Value	-0.052	-0.052	-0.052	-0.052
	(-0.23)	(-0.23)	(-0.23)	(-0.23)
Time Fixed Effects	Y	Y	Y	Y
Regional Fixed Effects	Y	Y	Y	Y
Cons	4.080***	4.079***	4.073***	4.091***
	(3.14)	(3.14)	(3.13)	(3.14)
N	125006	125006	125006	125006
R^2^	0.213	0.213	0.213	0.213

Note:

***, **, and * denote significance at the 1%, 5%, and 10% levels, respectively.

#### 4.3.2 Robustness test

*4*.*3*.*2*.*1 Replacing the explanatory variable*. The overall Bank Digital Transformation Index is divided into three dimensions: strategic digitalization, business digitalization, and management digitalization. This division allows for a detailed analysis of the impact of commercial banks’ digital transformation on household income across various dimensions. Columns (2), (3), and (4) of Table 2 show that enhancements in all three digitalization dimensions contribute to an increase in total household income, affirming the robustness of the research findings.

*4*.*3*.*2*.*2 Adding control variables*. The internet penetration rate in a region significantly influences the use of digital financial services. Consequently, the authors incorporate the internet penetration rate of the province (or city, district) where the household resides as a control variable to reduce the impact of unobservable factors on the empirical findings. As demonstrated in Column (1) of Table 3, the inclusion of this variable maintains consistency with the coefficient signs and significance levels of the baseline regression, thereby verifying the robustness of the results.

**Table 3:** Commercial bank digitalization and household income: Endogeneity test.

	(1)	(2)	(3)
	Adding Explanatory Variables	Instrumental Variable	
	Total Household Income	Overall Digitalization Index	Total Household Income
Overall Bank Digital Transformation Index	0.063***		0.005**
	(11.25)		(2.49)
Internet Penetration Rate	-25.111***		
	(-11.15)		
ln(Number of Post Offices at the End of 1984 × Proportion of Mobile Internet Users)		27.7098***	
		(379.52)	
Control Variables	Y	Y	Y
Time Fixed Effects	Y	Y	Y
Regional Fixed Effects	Y	Y	Y
N	125006	125006	125006
R^2^	0.213	0.986	0.213
F-Value		144037	

Note:

***, **, and * denote significance at the 1%, 5%, and 10% levels, respectively.

*4*.*3*.*2*.*3 Instrumental variable method*. The authors utilize the logarithm of the number of post and telecommunications offices at the end of 1984, multiplied by the proportion of mobile internet users, as an instrumental variable for the digital transformation of commercial banks (Huang et al., 2019) [43]. The number of post and telecommunications offices at the end of 1984 represents the historical level of informatization in the provinces where the households reside, suggesting that the degree of digitization in the banking sector is dependent on historical influences. Meanwhile, the proportion of mobile internet users indicates the level of digital acceptance among residents in the provinces where the households reside. The interaction of these two factors reflects not only the attitude and acceptance level of household heads towards new phenomena but also the ability of the regions to cope with the impact of informatization. Since the interaction term between the logarithm of the number of post and telecommunications offices at the end of 1984 and the proportion of mobile internet users is generally unrelated to household income conditions, it satisfies the requirement for exogeneity.

Columns (2) and (3) of Table 3 present the regression results using the two-stage least squares (2SLS) method. The first-stage results in Column (2) indicate that the instrumental variable is significantly and positively correlated with the Bank Digital Transformation Index, thereby confirming the relevance of the instrumental variable. Column (3) presents the results of the second-stage regression. After employing the instrumental variable, the results remain consistent with the baseline regression, suggesting that the digital transformation of commercial banks significantly boosts household income. The F-value in the first stage of the instrumental variable exceeds 10, effectively ruling out the weak instrument problem. Consequently, the research findings are robust.

#### 4.3.3 Mechanism analysis

Columns (1) to (4) of Table 4 present the results of the mechanism analysis. An increase in the Overall Bank Digital Transformation Index is observed to enhance household credit accessibility, promote inclusive development of regional digital finance, increase household entrepreneurial opportunities, and boost the purchase of household financial investment products. These findings support Hypotheses H1, H2, H3, and H4.

**Table 4:** Digital transformation of commercial banks and household income: Mechanism analysis.

	(1)	(2)	(3)	(4)
	Credit Accessibility	Digital Inclusive Finance Index	Household Entrepreneurship	Purchase of Financial Investment Products
Overall Bank Digital Transformation Index	0.008***	2.419***	0.002***	0.019***
	(3.65)	(428.07)	(2.80)	(3.63)
Control Variables	Y	Y	Y	Y
Time Fixed Effects	Y	Y	Y	Y
Regional Fixed Effects	Y	Y	Y	Y
Cons	-5.000**	-507.979***	-9.712***	-11.386**
	(-2.35)	(-102.82)	(-65.53)	(-2.40)
N	125006	125006	125006	125006
*R* ^2^	0.203	0.987	0.151	0.210

Note:

***, **, and * denote significance at the 1%, 5%, and 10% levels, respectively.

#### 4.3.4 Heterogeneity analysis

*4*.*3*.*4*.*1 Heterogeneity analysis of digital transformation levels of commercial banks*. Drawing from the approach by Zhao et al. (2023) [44], this study analyzes the digital transformation across various commercial banking institutions. Banks are categorized into two groups based on the average digital transformation index between 2013, 2015, 2017, and 2019: high and low levels of digital transformation. The digital transformation of commercial banks is characterized from three perspectives: ownership structure, listing status, and business geographical scope.

Results from Table 5, Panel A, show that state-owned and joint-stock banks belong to the high digitalization group, whereas the majority of city commercial banks fall into the low digitalization group, with state-owned and joint-stock banks experiencing greater digital growth than city commercial banks. According to the results from Table 5, Panel B, listed banks surpass unlisted banks in both the degree of digitalization and the rate of digital growth.

**Table 5:** Heterogeneity of digitalization among commercial banks.

	Low Digitalization	High Digitalization	Total	Average Digitalization in 2013	Average Digitalization in 2019	Growth Rate
Panel A.						
State-Owned Banks	0	5	5	73.349813	154.7078	81.358
Joint-Stock Banks	0	11	11	66.489043	134.6719	68.18288
City Commercial Banks	46	42	88	24.861701	88.26488	63.40317
Total	46	58	104	31.595752	96.36769	64.77193
Panel B.						
Always Listed	0	16	16	70.04455	144.4958	74.45126
Between 2013 and 2019	4	17	21	32.828458	104.7117	71.88321
Never Listed	42	25	67	22.027579	82.25912	60.23154
Total	46	58	104	31.595752	96.36769	64.77193
Panel C.						
National Banks	0	16	16	68.633034	140.9331	72.30011
Municipalities, Provincial Capitals, and Sub-Provincial Cities	11	29	40	31.94082	98.35827	66.41745
General Prefecture-Level Cities	35	13	48	18.962435	79.85372	60.89128
Total	46	58	104	31.595752	96.36769	64.77193

Note:

***, **, and * denote significance at the 1%, 5%, and 10% levels, respectively.

Results from Table 5, Panel C, reveal that all national commercial banks are in the high digitalization group. Most commercial banks in directly governed municipalities, provincial capitals, and sub-provincial cities also belong to this group, while only a few commercial banks in other prefecture-level cities are part of this group. Additionally, the rate of digital growth is higher among national and major city commercial banks compared to those in other prefecture-level cities. These findings highlight significant variations and disparities in digitalization levels among different banks.

*4*.*3*.*4*.*2 Heterogeneity analysis of household environment*. Table 6, Panel A, details the variations in the impact of commercial bank digitization on household income across diverse regions, areas with competitive financial markets, and regions with varying degrees of inclusive finance development. Columns (1), (2), and (3) of Panel A show that in the Eastern region, the overall index of commercial bank digitization does not significantly influence household income. Conversely, in the Central and Western regions, the overall index of commercial bank digitization significantly boosts household income, with the outcomes confirmed via inter-group coefficient difference tests. This can be attributed to the well-developed infrastructure and already elevated levels of bank digitization in the Eastern region, where additional advancements in commercial bank digitization have a negligible impact on household income. However, in the Central and Western regions of China, where the financial infrastructure is less developed, enhancing the digital transformation of commercial banks significantly boosts household income levels. Columns (4) and (5) of Panel A reveal that in provinces with high financial market competition, the overall index of commercial bank digitization does not significantly affect household income. Conversely, in provinces with low financial market competition, the overall index significantly raises household income at a 1% confidence level. This could be due to the already elevated levels of bank digitization in provinces with intense financial market competition, where further enhancements in digitization levels have a minimal effect on household income. Columns (6) and (7) indicate that in provinces with advanced development in digital inclusive finance, the overall index of commercial bank digitization does not significantly impact household income. However, in provinces with low levels of development in digital inclusive finance, the overall index significantly boosts household income at a 1% confidence level. This may be because, in provinces with advanced development in digital inclusive finance, the level of bank digitization is already elevated, and further improvements in digitization do not significantly affect household income.

**Table 6:** Impact of bank digital transformation on household income: Heterogeneity analysis.

Panel A					
	Region			Degree of Financial Market Competition	
	(1)	(2)	(3)	(4)	(5)
	Eastern Region	Central Region	Western Region	High Level of Financial Industry Competition	Low Level of Financial Industry Competition
Digitalization Composite Index	-0.004	0.004*	0.028***	-0.004	0.010***
	(-1.31)	(1.96)	(8.17)	(-1.24)	(6.13)
Control Variables	Y	Y	Y	Y	Y
Time Fixed Effects	Y	Y	Y	Y	Y
Regional Fixed Effects	Y	Y	Y	Y	Y
Cons	-4.208*	-0.831	8.296***	-3.072	0.275
	(-1.69)	(-0.29)	(2.71)	(-1.23)	(0.14)
N	59207	33856	31943	58526	66480
R^2^	0.211	0.195	0.199	0.212	0.194
Panel B					
	Urban-Rural			Income	
	(1)	(2)		(3)	(4)
	Urban	Rural		High-Income Households	Low-Income Households
Digitalization Composite Index	0.007***	0.015***		0.003**	0.009***
	(3.59)	(7.74)		(1.96)	(5.68)
Control Variables	Y	Y		Y	Y
Time Fixed Effects	Y	Y		Y	Y
Regional Fixed Effects	Y	Y		Y	Y
Cons	2.832*	7.899***		7.726***	4.740***
	(1.66)	(4.16)		(5.36)	(2.97)
N	87776	37230		37326	87680
R^2^	0.182	0.237		0.153	0.108

Note:

***, **, and * denote significance at the 1%, 5%, and 10% levels, respectively.

Table 6, Panel B, explores the urban-rural divide and income heterogeneity in the effects of commercial bank digitization on household income. The findings from columns (1) and (2) of Panel B suggest that in urban areas, the overall index of commercial bank digitization exerts a more substantial influence on household income than in rural areas. This disparity can be attributed to more developed financial infrastructure, enhanced financial accessibility, increased entrepreneurial opportunities, and higher financial literacy in urban compared to rural areas, leading to a lesser impact of digital transformation on household income through commercial banks. The findings from columns (3) and (4) of Panel B reveal that the impact coefficient of the overall index of commercial bank digitization on high-income households is less than that on low-income households. This may be due to high-income households possessing more wealth, which affords them greater opportunities for credit and access to financial products. Conversely, low-income groups are frequently excluded from the financial system. Enhancing the digital transformation of commercial banks can more effectively improve financial accessibility for low-income families, thus boosting household income (Liu et al., 2024) [45].

### 4.4 Further analysis

The earlier results demonstrate that the digital transformation of commercial banks has significantly boosted household income. Considering that total household income includes wage income, agricultural business income, industrial and commercial business income, property income, and transfer income, we further break it down to examine the impact of commercial bank digital transformation on these diverse income types. Columns (1) through (5) of Table 7 indicate that the digital transformation of commercial banks has significantly enhanced industrial and commercial business income, property income, and transfer income.

**Table 7:** Further analysis of the impact of commercial bank digitalization on household income.

	(1)	(2)	(3)	(4)	(5)
	Wage Income	Agricultural Business Income	Industrial and Commercial Business Income	Property Income	Transfer Income
Digitalization Composite Index	0.033	0.005	0.007***	0.018***	0.022***
	(0.76)	(1.20)	(3.02)	(7.00)	(7.50)
Control Variables	Y	Y	Y	Y	Y
Time Fixed Effects	Y	Y	Y	Y	Y
Regional Fixed Effects	Y	Y	Y	Y	Y
Cons	8.952**	-11.630***	-8.593***	-19.576***	8.322***
	(2.54)	(-4.22)	(-3.85)	(-7.91)	(2.99)
N	125006	125006	125006	125006	125006
*R* ^2^	0.266	0.213	0.089	0.198	0.205

Note:

***, **, and * denote significance at the 1%, 5%, and 10% levels, respectively.

## 5. Research conclusions and policy recommendations

### 5.1 Research conclusions

This study empirically confirms the impact of commercial bank digital transformation on household income, thereby enriching the existing literature on financial inclusion, digital finance, and household economics. It demonstrates how digital advancements improve the efficiency and accessibility of banks, thus economically benefiting households. The research highlights the multidimensional nature of digital transformation, encompassing strategic, operational, and managerial aspects, each contributing to the financial well-being of families. Furthermore, this paper explains how the digital transformation of commercial banks can reduce income inequality and foster inclusive development. Specifically in the Central and Western regions, areas with less financial sector competition, regions with lower levels of digital inclusive finance development, rural areas, and among low-income families, the impact of commercial bank digital transformation on increasing household income is especially significant.

### 5.2 Policy recommendations

#### 5.2.1 Government perspective

First, governments should offer tax incentives and subsidies to bolster investments in digital infrastructure in rural and other underdeveloped areas. Second, governments should implement regulations to ensure that banks treat all customers equitably during digital transformation, particularly those with low incomes and weak credit, in an effort to increase the inclusiveness of financial services. Third, governments should promote the development of financial technology regulatory sandboxes to assess the potential risks of new technologies. Additionally, governments should formulate contingency plans to manage potential credit tightening during economic downturns and ensure the stability of credit markets.

#### 5.2.2 Bank perspective

First, banks should utilize big data and artificial intelligence to more precisely assess customer credit risk, ensuring rational allocation of credit resources and enhancing services to SMEs and households. Second, banks should capitalize on their extensive branch network and rich human resources to bolster fintech research and development, accelerate the integration of digital technologies with credit allocation, and provide customized financial products and services to meet diverse customer needs. Third, banks should collaborate with fintech companies to advance the deep integration of financial services with the real economy. In scenarios of financial supply and demand imbalances, they should more effectively leverage the policy-oriented, targeted, and inclusive benefits of digital technologies and scientific methods.

#### 5.2.3 Household and individual perspective

First, individuals should improve their financial knowledge and skills, actively engage in financial education and training, and enhance their understanding and use of digital financial services. By leveraging digital services provided by banks, they can access loans and other financial services more conveniently, thereby alleviating issues related to financing difficulties and high costs. Second, individuals should remain vigilant while using digital financial services, safeguard against potential financial risks, manage their assets prudently, and avoid excessive borrowing and investment risks. Third, to ensure financial decisions are made within a safe and controlled environment, individuals should actively monitor and comprehend the financial policies and dynamics of the government and banks, improving financial literacy to facilitate wealth accumulation and risk management.

## Supporting information

S1 Data (DTA)
